# Skull vibration induced nystagmus, velocity storage and self-stability

**DOI:** 10.3389/fneur.2025.1533842

**Published:** 2025-02-04

**Authors:** Ian S. Curthoys, David S. Zee, Georges Dumas, Christopher J. Pastras, Julia Dlugaiczyk

**Affiliations:** ^1^Vestibular Research Laboratory, School of Psychology, The University of Sydney, Sydney, NSW, Australia; ^2^Departments of Neurology, Neuroscience, Ophthalmology, Otolaryngology-Head and Neck Surgery, The Johns Hopkins University School of Medicine, The Johns Hopkins Hospital, Baltimore, MD, United States; ^3^Department of Oto-Rhino-Laryngology Head and Neck Surgery, University Hospital, Grenoble, France; ^4^Research Unit DevAH — Development, Adaptation and Handicap, Faculty of Medicine, University of Lorraine, Vandoeuvre-lès-Nancy, France; ^5^Faculty of Science and Engineering, School of Engineering, Macquarie University, Sydney, NSW, Australia; ^6^Department of Otorhinolaryngology, Head and Neck Surgery & Interdisciplinary Center for Vertigo, Balance and Ocular Motor Disorders, University Hospital Zurich (USZ), University of Zurich (UZH), Zurich, Switzerland

**Keywords:** self-stability, utricular, vibration induced nystagmus, velocity storage integrator, self-motion, semicircular canal, tinnitus, vestibular

## Abstract

In this paper we give an introduction to the area, followed by brief reviews of the neural response to sound and vibration, and then the velocity storage integrator, before putting forward our hypothesis about the neural input to the velocity storage integrator. Finally we discuss some of the implications of our hypothesis. There are two pathways conveying neural information from the vestibular periphery (the semicircular canals and the otoliths) to central neural mechanisms—a direct and an indirect pathway. Within the indirect pathway there is a unique neural mechanism called the velocity storage integrator (VSI) which is part of a neural network generating prolonged nystagmus, afternystagmus and the sensation of self-motion and its converse self-stability. It is our hypothesis that only neural input from primary afferent neurons with irregular resting discharge projects in the direct pathway, whereas the primary afferent input in the indirect pathway consists of neurons with regular resting discharge. The basis for this hypothesis is that vibration is a selective stimulus for vestibular neurons with irregular resting discharge. 100 Hz mastoid vibration, while capable of generating nystagmus (skull vibration induced nystagmus SVIN), is ineffective in generating afternystagmus (in the condition of an encased labyrinth) which is a marker of the action of the VSI, leading to the conclusion that irregular afferents bypass the indirect pathway and the VSI. In order to present this hypothesis we review the evidence that irregular neurons are selectively activated by sound and vibration, whereas regular neurons are not so activated. There are close similarities between the temporal characteristics of the irregular afferent neural response to vibration and the temporal characteristics of SVIN. SVIN is a simple clinical indicator of whether a patient has an imbalance between the two vestibular labyrinths and our hypothesis ties SVIN to irregular primary vestibular neurons.

## Introduction

### Vibration as a vestibular stimulus

**Figure 1:**
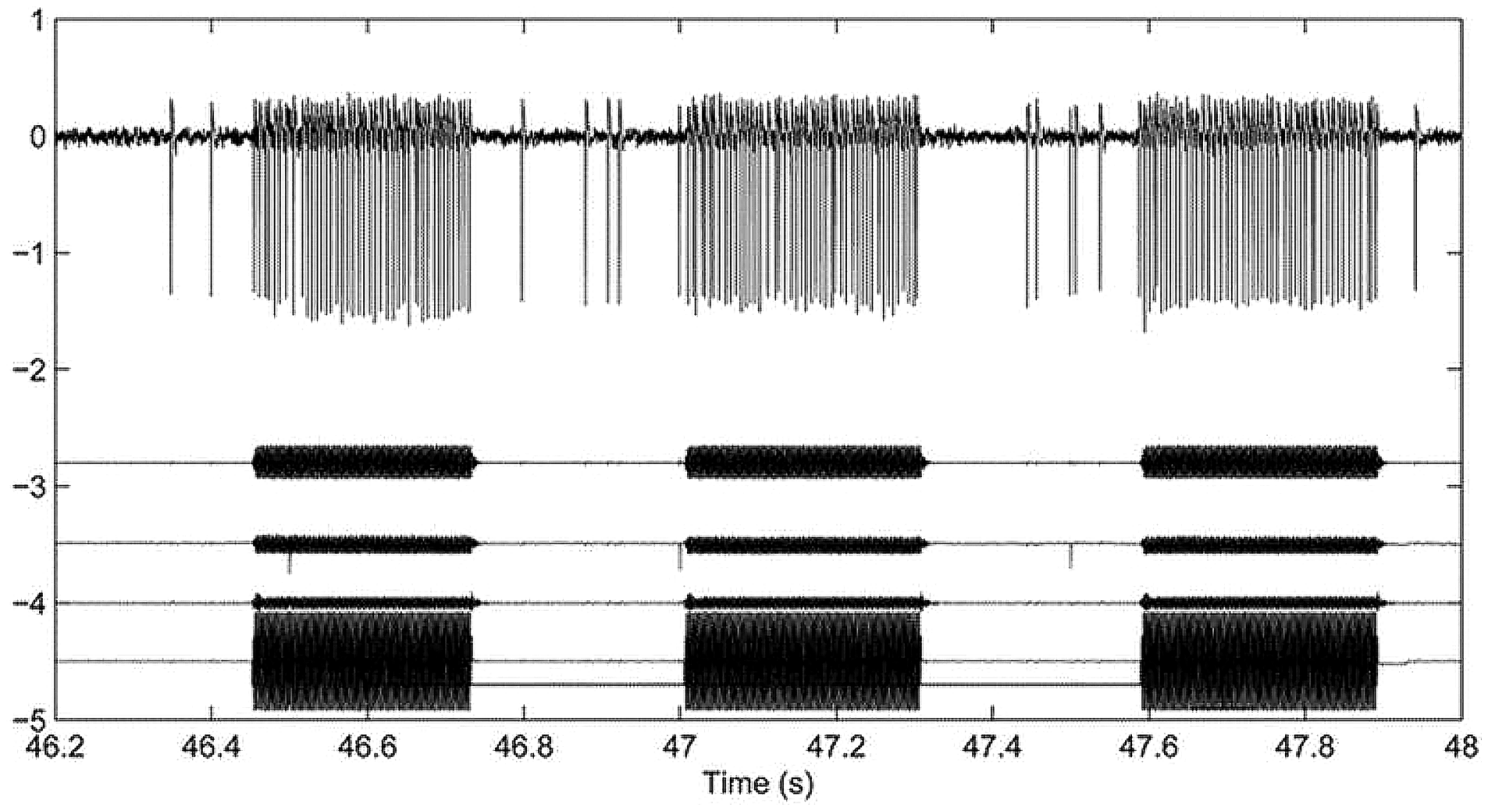
Top row: Action potentials in a guinea pig superior semicircular canal neuron after creating a superior canal dehiscence (SCD) in response to 3 bursts of 500 Hz bone conducted vibration. The action potentials (arbitrary voltage scale) are phase-locked to the stimulus (6). The records below the action potentials are 3 accelerometer traces (x, y, and z) and the command voltage. This shows the abrupt onset and offset of neural activation in response to tonal stimulation and the maintained firing during the stimulus. This is data replotted from Figure 3 of (8) with permission.

Recordings from identified primary vestibular neurons (both otolithic and canal) in anaesthetized guinea pigs with normally encased bony labyrinths show that bone conducted vibration (BCV) of the skull is an effective vestibular stimulus. It causes an increased firing rate selectively in vestibular neurons with irregular resting discharge, whereas BCV is largely ineffective in activating neurons with regular resting discharge (1–5). In animals with normally encased bony labyrinths, irregular otolithic afferents can be activated by BCV and phase lock up to very high frequencies >1,000 Hz (6). In such animals, irregular canal afferents can only be activated by BCV up to about 200 Hz, although after creating a dehiscence of the bony wall of the superior semicircular canal (an SCD), irregular semicircular canal afferents show activation and phase locking to much higher frequencies (>1,000 Hz) (7, 8). The receptors and irregular afferents which are activated by BCV have very similar anatomical and physiological aspects for both canals and otoliths—type I receptors enveloped by the calyx ending of a large diameter axon with irregular resting discharge. In the otoliths, irregular neurons originate from receptors at the striolae of the maculae (4, 9), and in the canals they originate from receptors at the crest of the crista (10). The timing of the action potentials of the BCV activated irregular neurons is phase-locked to the stimulus frequency (6, 7, 11) showing that each cycle of the BCV stimulus can generate an action potential in irregular neurons (Figure 1). Both otolithic and canal irregular neurons show phase locking which is evidence of the high temporal precision provided by the unique very rapid non-quantal transmission which appears to be required for precise phase-locking (12). In response to vibration, irregular neurons, both otolithic and canal, show a characteristic pattern of response (see Figure 1): abrupt onset and offset locked to the stimulus onset and offset with maintained firing with little adaptation for long-duration stimuli and no reversal at stimulus offset—the firing rate promptly returns to resting discharge at stimulus offset.

**Figure 2:**
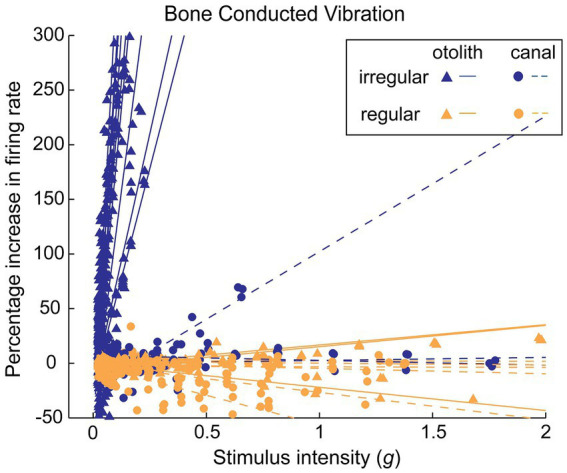
The differential response of irregular and regular primary otolithic guinea pig neurons to increasing levels of 500 Hz BCV stimulation. Each line shows the response of an individual neuron as stimulus intensity is increased, with data from many neurons superimposed in this one plot. Irregular neurons (blue lines) are strongly activated at very low intensities, whereas regular neurons (orange lines) show no activation even up to very high stimulus intensities. This is the evidence underpinning the different efficacy of BCV for regular and irregular neurons. Reproduced from (73) with permission.

It is important to note that primary neurons with regular resting discharge are not activated by the same BCV stimulus frequencies and amplitudes which activate irregular neurons. In sum, vibration is a selective stimulus for irregular vestibular afferents (Figure 2).

### SVIN—basic description

**Figure 3:**
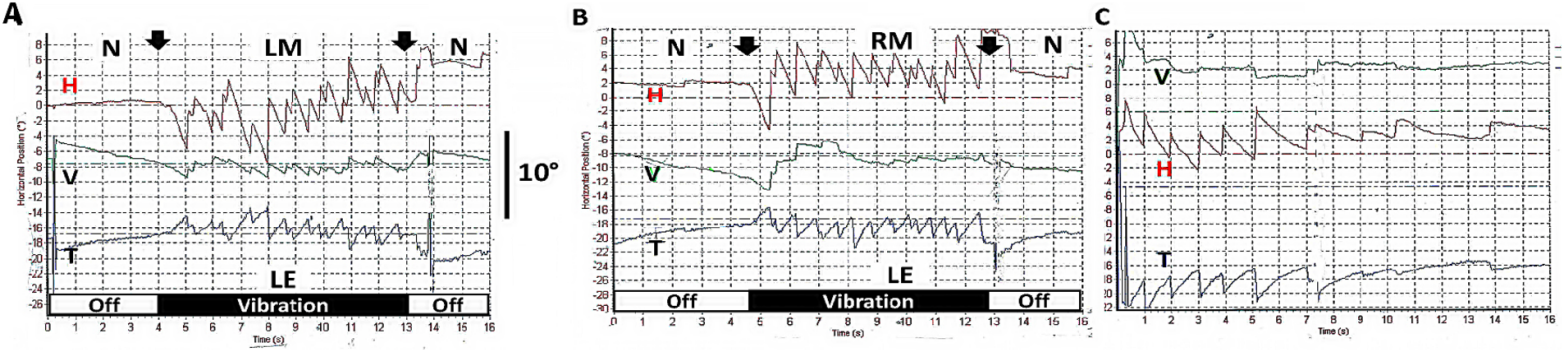
**(A,B)** Examples of skull vibration induced nystagmus in response to 100 Hz mastoid stimulation of each ear separately in a patient with complete left unilateral vestibular loss. **(A)** Left mastoid (LM). **(B)** Right mastoid (RM). H = horizontal, V = vertical, T = torsional nystagmus component. *N* = no stimulus applied. Eye movements were recorded from the left eye (LE). Note the abrupt nystagmus onset at vibration onset (first arrow), with little adaptation during the maintained BCV stimulus and the abrupt offset at stimulus termination (second arrow), with minimal afternystagmus. Vibration is a very different stimulus compared to rotational stimulation since each cycle of the vibration is acting to generate an action potential and so generates nystagmus immediately from stimulus onset and is maintained throughout the stimulus. Corresponding to that immediate onset and maintained neural firing, SVIN shows immediate onset and maintained slow phase eye velocity during the course of the stimulus, followed by an abrupt cessation of the nystagmus at offset with virtually no afternystagmus. In patients stimulated with 10 s vibration as here, an afternystagmus is only rarely observed and if present is only minimal (only 2 of 32 patients—6%) (74). **(C)** Head shaking nystagmus in the same patient (to be discussed below). Modified from data published in (74) with permission.

If patients with a complete unilateral vestibular loss are tested (with vision denied) by 100 Hz vibration of either mastoid, the result is a nystagmus called skull vibration induced nystagmus (SVIN) (13). The quick phases of this nystagmus beat toward the healthy side for vibration of either mastoid, almost certainly because the BCV is so efficiently transmitted through the skull that it activates the remaining irregular neurons, irrespective of the mastoid being stimulated (14). Unlike nystagmus due to rotation or caloric stimulation, SVIN starts with full slow phase eye velocity at BCV onset and is maintained with little adaptation during the maintained BCV stimulus and stops abruptly at BCV offset—with virtually no afternystagmus (Figure 3).

Healthy people with normal vestibular function tested with 100 Hz mastoid vibration usually show no consistent, systematic SVIN [above a threshold value for the slow phase eye velocity (SPV) of 2.5 deg./s] (13), probably because both labyrinths are stimulated simultaneously and about equally by the BCV of either mastoid and so the neural input from the two labyrinths effectively cancels each other at the vestibular nucleus. Such cancelation by simultaneous bilateral vestibular stimulation has been shown by Cohen et al. in responses to simultaneous high frequency electrical stimulation of both labyrinths in cats (15). However, in patients after a unilateral vestibular loss, the vibration induced neural activity from the healthy side will not be canceled by input from the affected side, resulting in an imbalance of neural activity between the two vestibular nuclei, which will generate nystagmus, just as the neural imbalance due to acute unilateral vestibular loss or rotation or caloric stimulation generates nystagmus (13, 14). As such SVIN is a simple clinical indicator of an imbalance between the two vestibular labyrinths (13, 14). Recently, Shemesh et al. have shown that the diagnostic value of this simple clinical indicator can be enhanced by comparing SVIN with the head in different orientations relative to gravity (right ear down versus left ear down) (16)—the affected ear down eliciting stronger nystagmus.

Our premise is that SVIN is due to the selective activation of vestibular afferents with irregular resting discharge by BCV (17). The immediate onset and immediate offset of the nystagmus with little or no afternystagmus differentiates SVIN from caloric or rotational nystagmus and raises major questions about how SVIN is generated centrally, especially in relation to how other forms of nystagmus are generated. The absence of afternystagmus is of particular importance as we explain below. In order to elaborate our hypothesis, we need to consider central mechanisms for nystagmus generation, including velocity storage. The following is a very brief summary of the relevant aspects of the velocity storage literature [for full recent reviews; see (18–20)].

### Velocity storage background

**Figure 4:**
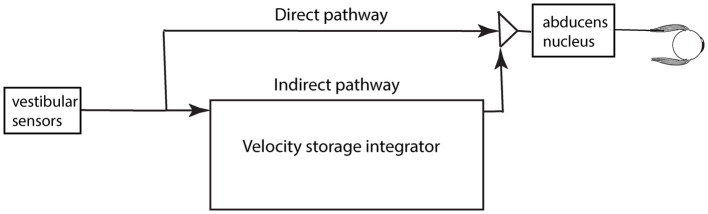
A schematic representation of the direct and indirect pathways conveying vestibular neural input centrally. The pathways and boxes represent neural networks in the brainstem at the level of the vestibular nuclei. Within the indirect pathway there is a network of neurons called the velocity storage integrator (VSI) which we discuss below.

The usual explanation of vestibular nystagmus generation contends that there are two parallel pathways—a direct and an indirect pathway—from the vestibular periphery to central structures which generate nystagmus (Figure 4) (21). The direct pathway is responsible for fast compensatory responses, for example the rapid compensatory eye movement to a brief abrupt head turn, e.g., in the head impulse test (22). The indirect pathway contains a neural network called the velocity storage integrator (VSI) which, as we explain more fully below, is held to enhance canal neural responses to low frequency stimulation and acts to prolong per-rotatory nystagmus and to cause afternystagmus when the vestibular stimulus is removed. The following is a brief review of the velocity storage integrator.

### Velocity storage origin

**Figure 5:**
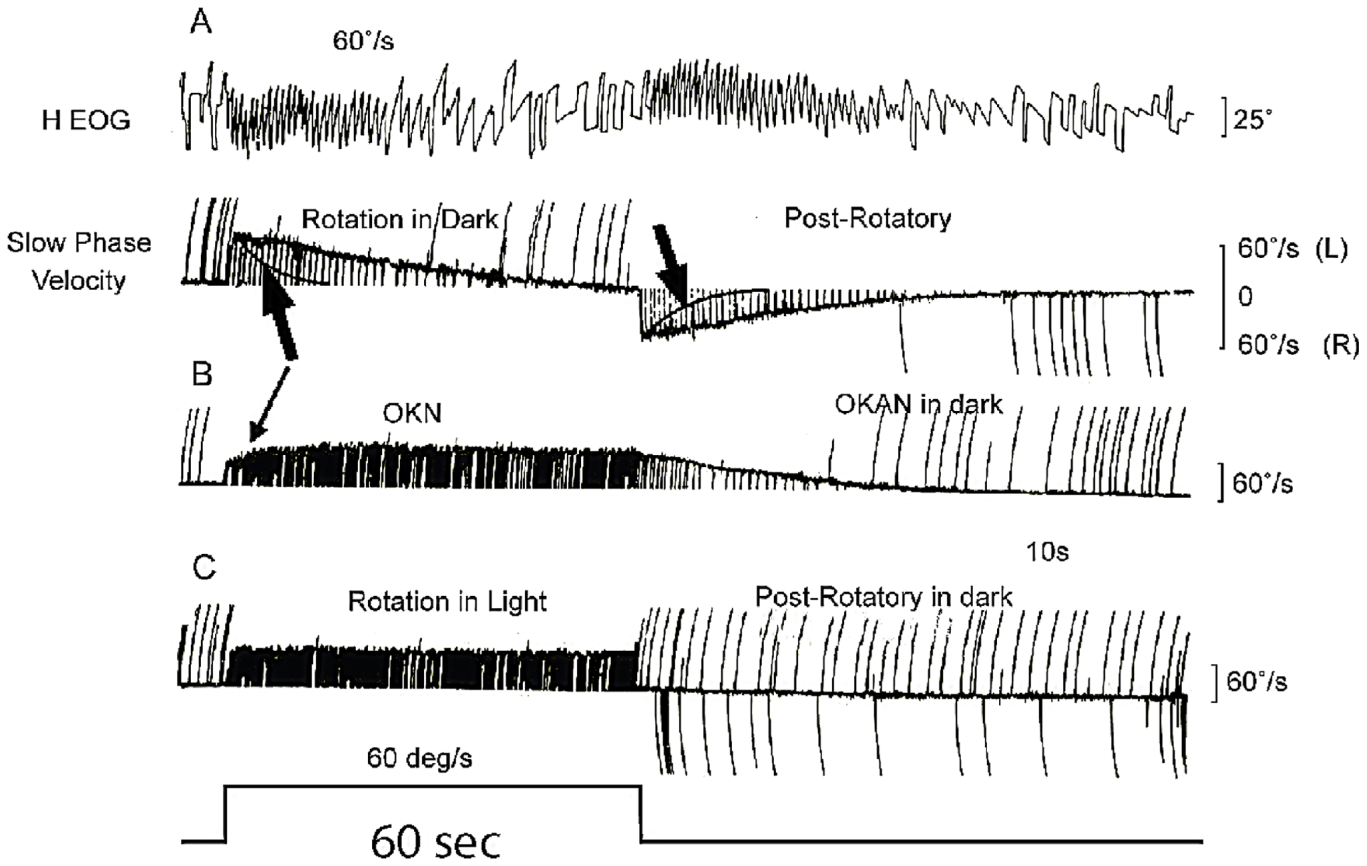
**(A)** The very long decay of post-rotatory vestibular nystagmus and the similar long decay of optokinetic nystagmus after stimulus offset when measured in darkness. The large black arrows in **(A)** point to the exponential cupula decay profile, showing the cupula has returned to rest (i.e., no canal activation) long before the afternystagmus declines. **(B)** Optokinetic nystagmus (OKN) shows similar slow decay (optokinetic afternystagmus OKAN). The time scale is shown by the stimulus step in the last trace—which is 60 s long. Reproduced from (23) with permission of Elsevier. **(C)** After rotation in full light, the post-rotatory nystagmus is cancelled by optokinetic afternystagmus.

The original idea for velocity storage came from the nystagmus results of monkeys given a yaw angular acceleration in total darkness. This stimulus caused a very long duration horizontal nystagmus (23), of much longer duration than expected, given knowledge of canal-cupula mechanics (24, 25). This long duration was unexpected since deflection of the cupula activates receptors and afferent neurons and causes nystagmus; but at constant velocity the cupula slowly returns to its resting position due to its own internal cupula elastic restoring force (24, 25) and thus at the end of the acceleration the receptors slowly return to their resting discharge (26), and as they do so the activation of the primary afferents declines. Cupula mechanics shows that the time constant of cupula return is about 4 s in humans (and about the same in monkeys) (27). After about 3 time constants, (i.e., about 12 s), the cupula has come to rest and no receptors are deflected, yet the nystagmus continues for a very long time after that (see Figure 5). It is as if the cupula is still slowly returning to rest (see arrows in Figure 5A). The puzzle is: why does the nystagmus continue for such a long time after the cupula has returned to rest, as there is no activation of the primary canal afferents to drive the nystagmus? The time constant of cupula return is about 4 s whereas the time constant of nystagmus decay is about 15–30 s.

Clearly it is not just the semicircular canal receptors and afferents driving the nystagmus response—there must be some other central neural network which continues to be active after the end of the stimulus in order to generate this very long duration nystagmus response after the cupula has returned to rest. Raphan and Cohen postulated that the prolonged nystagmus response was due to the slow “discharge” of a neural network in the indirect pathway in the brainstem and cerebellum which they called the “velocity storage integrator” (VSI). They argued that the VSI is, by virtue of internal neural interconnections, responsible for perseveration of canal (and also optokinetic) neural activity: the angular acceleration stimulus “charges” the velocity storage network and the slow nystagmus decline at stimulus offset was due to the VSI slowly discharging. Optokinetic input operates similarly—charging the VSI and causing optokinetic afternystagmus (OKAN) at optokinetic stimulus offset (Figure 5B). The network of neurons comprising the VSI includes the vestibular nuclei (28), the nodulus and uvula of the cerebellum (29–31), and the nucleus prepositus hypoglossi (18, 32). Within this network GABA_B_ is a major transmitter since the GABA_B_ agonist baclofen degrades velocity storage (33).

**Figure 6:**
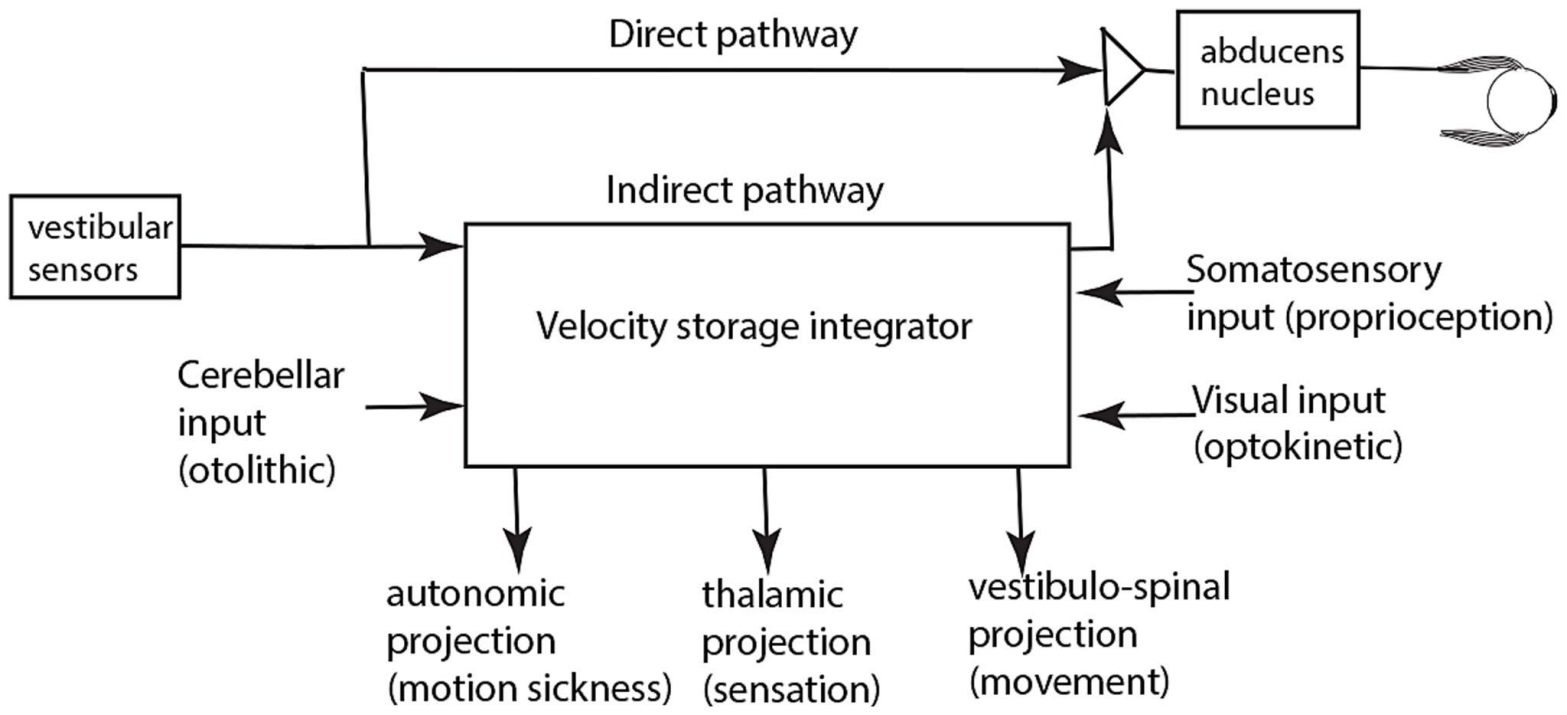
A simplified schematic summary of the inputs and outputs of the velocity storage integrator. It is based on published evidence referred to in the reviews specified above.

Over many years Cohen and Raphan and their students and colleagues conducted many experiments identifying how the VSI operates and they modeled its response [see (18, 34–37) for reviews]. The evidence from that research has shown that the velocity storage network receives neural input from many other systems apart from semicircular canal afferents—otolithic, visual, proprioceptive inputs—and projects to many other neural complexes apart from eye muscles (18, 34, 35, 37). Figure 6 summarizes in a very simplified schematic the major inputs and outputs for the VSI. Some anatomical results confirm the division of direct and indirect pathways—after selective section of the commissural fibers between the two vestibular nuclei, vestibular functions attributable to velocity storage were abolished, whereas the direct angular vestibulo-ocular reflex pathway remained intact (38).

Most importantly, as a result of a variety of experiments on human observers (see below) this velocity storage network is now recognized as being responsible not only for prolonged nystagmus responses but also for a fundamental sensory experience—the sensation of self-motion and its converse—self-stability. So, the velocity storage network not only generates prolonged nystagmus but also the fundamental sensation of self-stability and thus is central for understanding vertigo, postural and gait instability and also motion sickness (39, 40), by virtue of its neural projections to nuclei governing posture and autonomic responses [see reviews (18, 38)]. This account is for just the horizontal plane, but Laurens and Angelaki have emphasized the velocity storage integrator operates in three dimensions and ensures gaze stability when the head changes its orientation relative to gravity (41, 42). Shemesh et al. used such a 3D model to explain the effect of head orientation on SVIN in unilateral vestibular loss patients. Key to their interpretation was the assumption that the afferent activity from irregular primary afferents bypasses the velocity storage integrator (16). Thus, Shemesh et al. explain the effect of head orientation on SVIN in unilateral loss patients without using the velocity storage integrator (16).

### The function of velocity storage

This has been one of the most vexed questions in vestibular research. Raphan and Cohen argued that velocity storage enhances the very low frequency response of the semicircular canals: “By storing or integrating the signal coming from the cupula, the storage mechanism gives a more faithful representation of head velocity during slow head movement than the cupula signal itself” [(21), p. 244]. But even in the original 1979 paper Raphan et al. foreshadowed the role of velocity storage in self-motion perception. They state: “Consequently, monitoring of activity from the integrator as well as the peripheral vestibular apparatus and the visual system could be important for perception of self and environmental motion” [(21), p. 244]. Self-motion is now widely used in studying heading direction during active (or passive) locomotion, e.g., (43), but here we wish to use the term to denote the very simple idea of movement of the self (and its important converse self-stability).

Large moving visual stimuli (optokinetic stimuli) have strong input to the velocity storage network (44) and generate nystagmus and afternystagmus (Figure 5) and also sensations of self-motion (vection) in human subjects, as is clear from the illusory self-motion experienced in a stationary train as the adjacent train moves: there is an overpowering sensation of linear self-motion although peripheral vestibular and somatosensory input both signal that the subject is not moving. But in this situation the optokinetic visual input determines the self-motion perception, and it is argued that it does so because the VSI receives optokinetic input (36, 45, 46) (Figure 6) and this optokinetic input is causing the sensation of self-motion.

**Figure 7:**
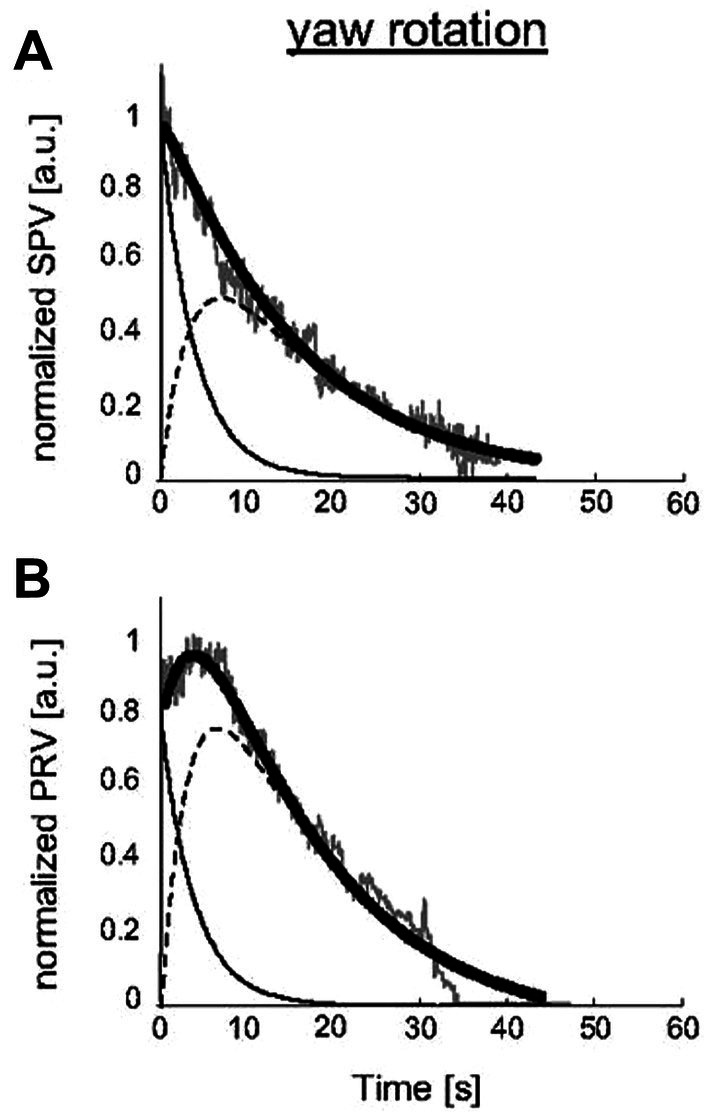
The close correspondence in human subjects between slow phase eye velocity (SPV) decline after angular acceleration **(A)** and the decline of perceived rotational velocity (PRV) **(B)**, both measured in arbitrary units (a.u.). Gray traces: SPV and PRV traces; black thick traces: SPV and PRV fits; thin black traces: semicircular canal contribution; dashed black traces: velocity storage mechanism [reproduced with permission from (48)].

The close connection between velocity storage and self-motion has been demonstrated by experiments on healthy human subjects measuring nystagmus and perceived self-motion during semicircular canal stimulation. Bertolini et al. confirmed an earlier demonstration by Okada et al. (47) of the close correspondence between the decay of rotational nystagmus and the decay of perceived self-motion (48). Bertolini et al. duplicated the original monkey experiment of (21) described above, but using humans rather than monkeys as subjects. They gave healthy human subjects an abrupt step of velocity in total darkness and measured the post-rotatory nystagmus and its decay. However, as well as measuring nystagmus they simultaneously measured the subject’s sensation of rotation by requiring the subject to turn a tachometer to indicate the velocity of their self-motion. Figure 7 shows the results, and it is clear that the perception of self-motion has a very long decay after the termination of the angular acceleration stimulus, closely corresponding to the long decay of post-rotatory nystagmus. Bertolini et al. concluded that the VSI determines not only nystagmus but also perception of self-motion. They argued that the angular acceleration charged the velocity storage network, followed by the slow decay as the network was discharged. That conclusion is now widely accepted: it is recognized that a major role of velocity storage is to generate the sensation of self-motion and orientation (43, 49).

How can this indirect pathway and the VSI be assessed in the clinic? There is now abundant evidence that after unilateral vestibular loss patients show responses which are acknowledged as being due to the operation of the velocity storage integrator, specifically head shaking nystagmus (50, 51). The person is given vigorous horizontal head shaking (for 15 s at 2 Hz) and at the cessation of that shaking, the nystagmus caused by that stimulus is referred to as head shaking nystagmus (HSN; Figure 3C) (51, 52). Such head shaking causes the cupula to be deflected back and forth, and in patients with unilateral vestibular loss their neural asymmetry is sufficient to charge the VSI and cause an afternystagmus at the end of the head shaking. Unilateral vestibular loss patients show head shaking nystagmus whereas healthy subjects do not, probably because both labyrinths are activated successively so the VSI is not charged. The presence of head shaking nystagmus is now recognized as a clinical test of the indirect pathway and the VSI (18). Some have used it as an index of vestibular compensation after unilateral loss—so it is reported that as compensation progresses the HSN decreases (53, 54). The nystagmus after head shaking is in sharp contrast to the absence of nystagmus after comparable vibration stimulation.

**Figure 8:**
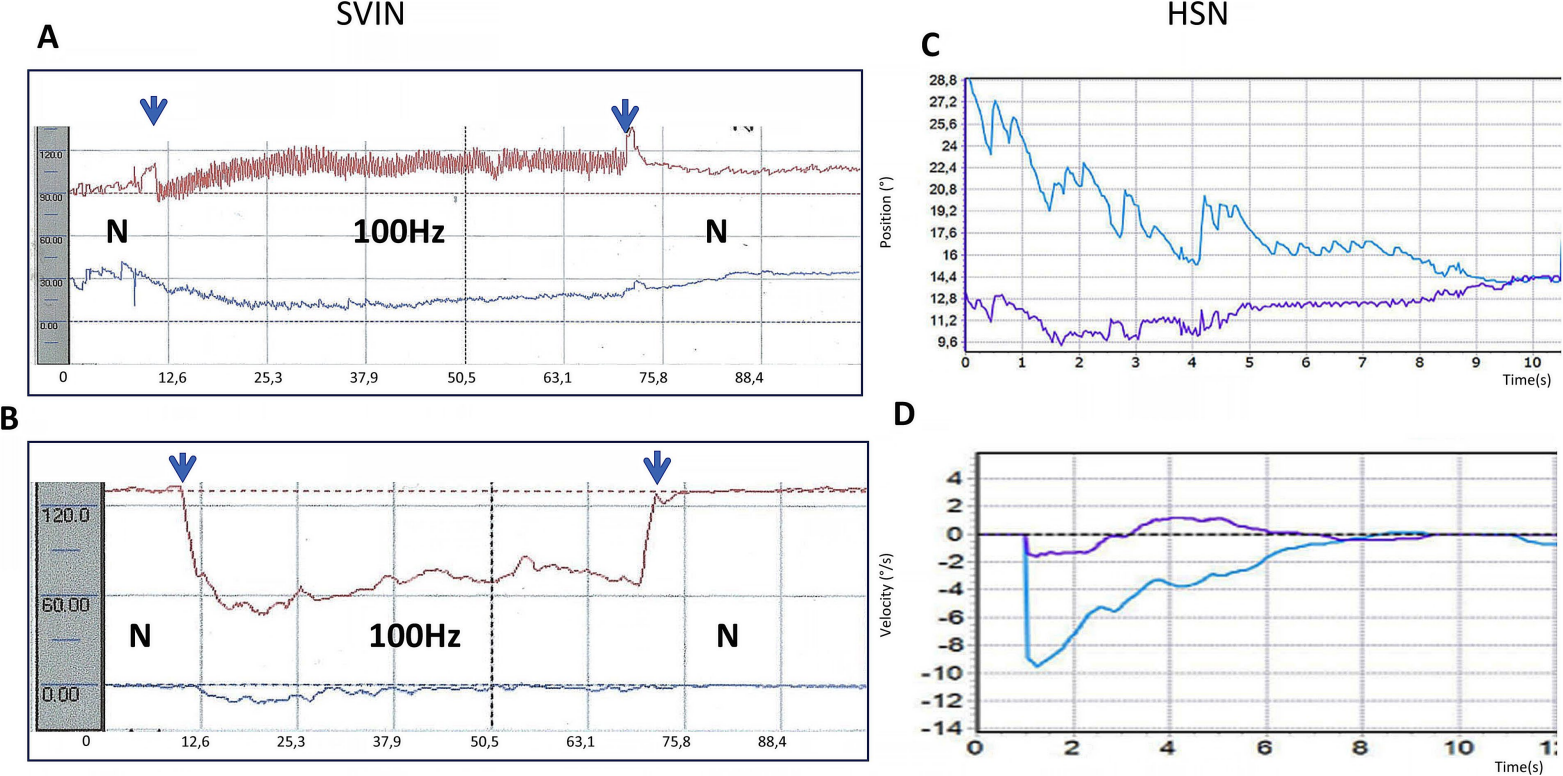
**(A,B)** Skull vibration induced nystagmus (SVIN). **(A)** Time series of eye position in response to 100 Hz vibration stimulation of the right mastoid for 60 s. in a patient with left unilateral vestibular loss. **(B)** Shows corresponding eye velocity with little adaptation during stimulation. *N* = no stimulus applied. The records show the abrupt onset and offset and maintained nystagmus during this long-duration 100 Hz stimulation. Note the virtual absence of afternystagmus at the end of stimulation (arrow) even after such a prolonged vibration stimulus. **(C,D)** The results of head shaking nystagmus (HSN) in this same patient, demonstrating that the patient does have HSN and thus their velocity storage integrator is functional (see also Figure 3C above where the patient also has SVIN without afternystagmus, but does have head shaking nystagmus confirming the presence of the velocity storage integrator in both patients, but in both cases SVIN does not generate afternystagmus).

SVIN has three very interesting aspects which differentiate it from nystagmus due to rotation or caloric stimulation (Figure 8) (13). They are: (1) SVIN has an abrupt onset at stimulus onset and (2) is maintained during vibration stimulation and (3) at stimulus termination the nystagmus ceases abruptly and (4) there is little or no afternystagmus after the end of the vibration. Clinical testing with both short-duration and long-duration 100 Hz mastoid vibration stimulation of patients with unilateral vestibular loss shows there is usually little or no evidence of the prolonged afternystagmus that occurs after the cessation of the vibration stimulus as compared to the decay in head shaking nystagmus.

A major difference between SVIN and post-rotatory nystagmus due to angular acceleration is that angular acceleration activates both regular and irregular primary semicircular canal neurons. However, as we have explained above, 100 Hz vibration selectively activates primary neurons with irregular resting discharge, both otolithic (to very high frequencies) and irregular semicircular canal neurons (to about 200 Hz) (7, 8). The activation of single irregular neurons shows little evidence of maintained discharge after stimulation offset (7) (Figure 1). Neurons with regular resting discharge are not usually activated by sound (55, 56) or vibration (1) (Figure 2).

## Hypothesis

To account for the very different afternystagmus responses to skull vibration as opposed to low frequency semicircular canal stimulation we now put forward the hypothesis that:

“The neural response to bone conducted vibration bypasses the indirect pathway and the velocity storage integrator.”

That hypothesis has major implications for clinical vestibular testing—that stimuli which largely stimulate irregular afferents (e.g., vibration, head impulse stimuli) appear to bypass the velocity storage integrator and so do not assess the status of the central network responsible for the subjective experience of self-motion (vertigo). In other words: the standard instrumental clinical tests of high frequency peripheral vestibular function (e.g., video head impulse test, vestibular evoked myogenic potential tests, SVIN), while valuable for providing information about the level of function of peripheral vestibular receptors by driving the direct pathway, do not address the major cause of the patient’s complaints of vertigo and lack of self-stability which arise from the VSI. The indirect pathway is properly assessed by stimulation such as low frequency rotation or caloric stimulation or head shaking nystagmus, all of which stimulate regular afferent neurons. Most patients with complete unilateral vestibular loss receiving SVIN do not experience self-motion or vertigo, but other patients do, when testing involves the indirect pathway—as shown by their response to head shaking nystagmus.

As we have argued SVIN is due to the activity of irregular neurons, so the corollary is that the usual means of charging the velocity storage integrator (i.e., by caloric or rotational stimulation) is via input from primary afferent neurons with regular resting discharge. On this account, calorics and rotation activate both types of neurons—those with irregular as well as those with regular resting discharge—and so activate both the direct and indirect pathways, including the VSI, and result in afternystagmus. On this account it follows that the afternystagmus to long-duration angular acceleration stimulation is due to velocity storage in the indirect pathway, originating from the activity of primary neurons with regular resting activity. Physiological data is consistent with the hypothesis that irregular neurons do not contribute to the VSI. Minor and Goldberg reported that using galvanic stimulation to silence irregular afferents (and so to silence their input to the direct pathway in our terms) had no effect on canal responses to low frequency rotational stimulation (57). Recently Ono et al. reported that mutant mice without type I receptors (and thus probably without irregular afferents) had absent responses to transient stimuli (vestibular sensory evoked potentials, VsEP) but normal canal induced responses—angular vestibulo-ocular response and off-vertical axis rotation responses (58). That result is consistent with the hypothesis that the input to the indirect pathway and the velocity storage integrator is from regular afferents and that irregular afferents bypass the velocity storage integrator.

The absence of afternystagmus after skull vibration in patients with unilateral vestibular loss may be due to the fact that VSI in these patients is absent or compromised such that the vibration stimulus is ineffectual in charging the VSI. However the same patients who show little or no afternystagmus to vibration (even long-duration vibration; Figure 8), often still show afternystagmus after head shaking (HSN) (13) implying that their velocity storage integrator is still functioning. In other words: these patients do have a functioning VSI sufficient to generate head shaking nystagmus if the peripheral vestibular stimulus is appropriate. So, the VSI is present in these patients, but not effective for BCV stimulation, since there is minimal afternystagmus to mastoid vibration.

Why should there be such a difference between the nystagmus due to vibration and that due to head shaking? During head shaking, both regular and irregular afferent neurons are activated, whereas with skull vibration it is almost exclusively irregular neurons only which are activated.

These results support our conclusion that it is peripheral afferents with *regular* resting discharge which provide input to the indirect pathway and the VSI. These receptors and afferents with regular resting discharge will be activated by head shaking but not by mastoid vibration. A direct test of our hypothesis is that baclofen should reduce HSN but have little effect on SVIN, because baclofen has been shown to reduce measures of velocity storage (33).

### Other considerations—SVIN in SCD

As noted above, Dumas has tested patients who have total unilateral vestibular loss with 100 Hz mastoid vibration stimuli lasting up to 90 s and found very little afternystagmus (Figure 8). On the other hand, Dumas (and others) found SVIN and afternystagmus in response to skull vibration in 25% of patients with SCD (59) as well as other signs of velocity storage (head shaking nystagmus). So the presence of afternystagmus in these SCD patients implies that 100 Hz vibration can access the velocity storage integrator.

What could cause those apparently inconsistent results? The evidence from Iversen and Rabbitt (60, 61) shows that in a semicircular canal with an SCD, vibration induces endolymph flow which deflects the cupula and so activates irregular neurons and also regular neurons and thus activates velocity storage. Curthoys indirectly confirmed endolymph flow after SCD: by recording from regular neurons in guinea pigs with an artificial SCD and testing their response to long-duration vibration stimulation. It was found there was a slow systematic decrease in the firing rate of the regular neurons over many seconds during vibration stimulation. This was attributed to the steady fluid flow which Iversen and Rabbitt had measured, deflecting the cupula and resulting in the slow decrease in the firing rate of the regular neuron [see Figure 3 of (62)]. Other regular neurons in these SCD animals showed a slow increase in firing rate during vibration. After vibration offset there was a slow return to resting discharge, presumably reflecting the cupula return to its resting position, driven by its elastic restoring force. So we suggest that the presence of afternystagmus in SCD patients is likely due to the fact that the cupula has been displaced by endolymph flow as well as by the cycle-by-cycle receptor activation in response to vibration (62). Such cupula displacement activates both regular and irregular afferents and so activates the indirect pathway and the VSI.

### SVIN and vision

As noted above vision partially suppresses SVIN (13, 63), so how can that be reconciled with the model put forward above where visual input projects to the velocity storage integrator in the indirect pathway? The answer is that neurons in the direct pathway also have their vestibular activity suppressed by vision (64–67) probably by means of cerebellar inhibition.

### Information theory

The activity of irregular and regular neurons has been the subject of a different research perspective from an information-theoretic point of view, studying the relative information transmitted by regular as opposed to irregular primary afferents and their relative roles in processing active or passive angular acceleration stimulation (68–70). One conclusion from that work is that regular neurons convey information using rate coding, whereas irregular neurons convey information using precise spike timing (71, 72). The hypothesis and argument presented here are in close agreement with that conclusion. Rather than information transmission we have focused on the roles of irregular vs. regular neurons in the generation of nystagmus in response to bone-conducted vibration.

### Auditory storage

In evolutionary terms the cochlea is derived from the vestibular system and an interesting question is: is there auditory storage analogous to velocity storage in the vestibular system? Why would nature erase velocity storage in the development of the auditory system—a newer system in an evolutionary sense. Is it due to the fact that what was needed was a high precision system for detecting transient events- the cycle-by-cycle stimuli in the auditory system—without needing to deal with prolonged stimulation, like gravity etc.? It appears that the direct pathway may be the natural precursor of the auditory system. One argument is that auditory storage analogous to velocity storage would defeat the main objective of auditory processing which is high fidelity, fast temporal processing and signaling. Neural perseveration in response to an auditory stimulus would act in precise opposition to such an objective. Nevertheless there are close parallels between aspects of the neural coding by vestibular irregular afferents and auditory afferent neurons—for example, phase locking shows the precision of these vestibular irregular neurons which are temporal detectors which can give information about individual cycles of the stimulus. Another argument is that perseveration of the neural response in the auditory system would be disastrous, but possibly tinnitus may be due to such inappropriate auditory perseveration.

## Overall

Our position is that the velocity storage integrator is a major determinant of the subjective sensation of self-motion and its converse self-stability. It is responsible for the sensation of being “anchored”—the sensation that you are located where you are. It is this sensation which, in many vestibular patients, is dysfunctional—they complain of postural and gait instability, of feeling that they are on a rocking boat. We contend that it is primary neurons with regular resting discharge which preferentially project to the VSI and so their dysfunction is primarily responsible for these patient reports of self instability. Superimposed on this stable “anchor” are the transient perturbations during rapid head movements which require rapid compensatory responses, but which pose a minimal challenge to the sustained sense of self-stability. For example, in response to a brief head impulse there is a compensatory eye movement response, but subjects do not experience a sensation of self-motion to such a transient stimulus. Driven by irregular neurons during the impulse in video head impulse testing, the seated subject’s eyes move to correct for the transient head movement, but the subject still retain their self-stability. We suggest that responses driven by the fast compensatory direct pathway with input from irregular afferents are not effective in charging the velocity storage integrator and so the feeling of self-stability is less affected. It appears that this subgroup of afferents in the vestibular nerve—irregular afferents—have the same goal as hearing has—for detecting and responding to transient events—but in the vestibular system there is an additional, parallel, much slower “anchor” mechanism. One conjecture is that when we actively explore the world, we will need information from the irregular afferents about quick (high frequency, and maybe even high acceleration) perturbations, whereas when we want a stable platform, the perturbations we must usually compensate for are lower frequency, so we need our velocity storage mechanism to provide sustained stability of gaze.

## Conclusion

There are two parallel pathways governing vestibulo-ocular responses, the direct and indirect pathways feeding through the vestibular nuclei and cerebellum out to effectors such as eye muscles. The direct pathway is responsible for rapid compensatory eye movements to brief high-acceleration stimulation whereas the indirect pathway is responsible for the slow nystagmus response and the sensation of self-motion. Our hypothesis is that it is irregular primary afferents which supply the direct pathway and bypass the indirect pathway and the velocity storage integrator. There is little evidence of velocity storage in SVIN—in particular prolonged afternystagmus is absent. We contend that the high acceleration stimuli which require rapid corrections by oculomotor and postural responses are accomplished by signaling in the direct pathway with minimum signaling by the indirect pathway and so minimum impact on self-motion or self-stability. These responses to transient demands are very different from the responses to sustained input from regular neurons generating a perception of self-stability.

## Author’s note

We dedicate this paper to the memory of Hans Straka. We pay tribute to the many insightful contributions he made to the understanding of vestibular function. His extraordinary breadth of knowledge, his scholarship, his drive, his collegiality, his skills in excruciatingly difficult electrophysiological experiments, his ideas—always asking the next question and then testing it—are a marvelous example of how to conduct vestibular research. His contributions and his approach to the study of this complex system is a continuing beacon to everyone in vestibular research and scientific endeavor generally.

## Data Availability

The original contributions presented in the study are included in the article/supplementary material, further inquiries can be directed to the corresponding author.
